# Serum Complement Activation by C4BP-IgM Fusion Protein Can Restore Susceptibility to Antibiotics in *Neisseria gonorrhoeae*

**DOI:** 10.3389/fimmu.2021.726801

**Published:** 2021-09-01

**Authors:** Serena Bettoni, Karolina Maziarz, M Rhia L Stone, Mark A T Blaskovich, Jan Potempa, Maria Luiza Bazzo, Magnus Unemo, Sanjay Ram, Anna M. Blom

**Affiliations:** ^1^Department of Translational Medicine, Lund University, Malmö, Sweden; ^2^Centre for Superbug Solutions, Institute for Molecular Bioscience, The University of Queensland, Brisbane, QLD, Australia; ^3^Faculty of Biochemistry, Biophysics and Biotechnology, Jagiellonian University, Kraków, Poland; ^4^Department of Oral Immunity and Infectious Diseases, University of Louisville School of Dentistry, Louisville, KY, United States; ^5^Molecular Biology, Microbiology and Serology Laboratory, Federal University of Santa Catarina, Florianópolis, Brazil; ^6^World Health Organization (WHO) Collaborating Centre for Gonorrhoea and other STIs, Department of Laboratory Medicine, Örebro University, Örebro, Sweden; ^7^Department of Medicine, Division of Infectious Diseases, University of Massachusetts Medical School, Worcester, MA, United States

**Keywords:** complement, antibiotic resisitance, *Neisseria gonorrhoeae*, C4b binding protein, membrane attack complex (MAC)

## Abstract

*Neisseria gonorrhoeae* is the etiological agent of gonorrhea, the second most common bacterial sexually transmitted infection worldwide. Reproductive sequelae of gonorrhea include infertility, ectopic pregnancy and chronic pelvic pain. Most antibiotics currently in clinical use have been rendered ineffective due to the rapid spread of antimicrobial resistance among gonococci. The developmental pipeline of new antibiotics is sparse and novel therapeutic approaches are urgently needed. Previously, we utilized the ability of *N. gonorrhoeae* to bind the complement inhibitor C4b-binding protein (C4BP) to evade killing by human complement to design a chimeric protein that linked the two N-terminal gonococcal binding domains of C4BP with the Fc domain of IgM. The resulting molecule, C4BP-IgM, enhanced complement-mediated killing of gonococci. Here we show that C4BP-IgM induced membrane perturbation through complement deposition and membrane attack complex pore insertion facilitates the access of antibiotics to their intracellular targets. Consequently, bacteria become more susceptible to killing by antibiotics. Remarkably, C4BP-IgM restored susceptibility to azithromycin of two azithromycin-resistant clinical gonococcal strains because of overexpression of the MtrC-MtrD-MtrE efflux pump. Our data show that complement activation can potentiate activity of antibiotics and suggest a role for C4BP-IgM as an adjuvant for antibiotic treatment of drug-resistant gonorrhea.

**Graphical Abstract:**
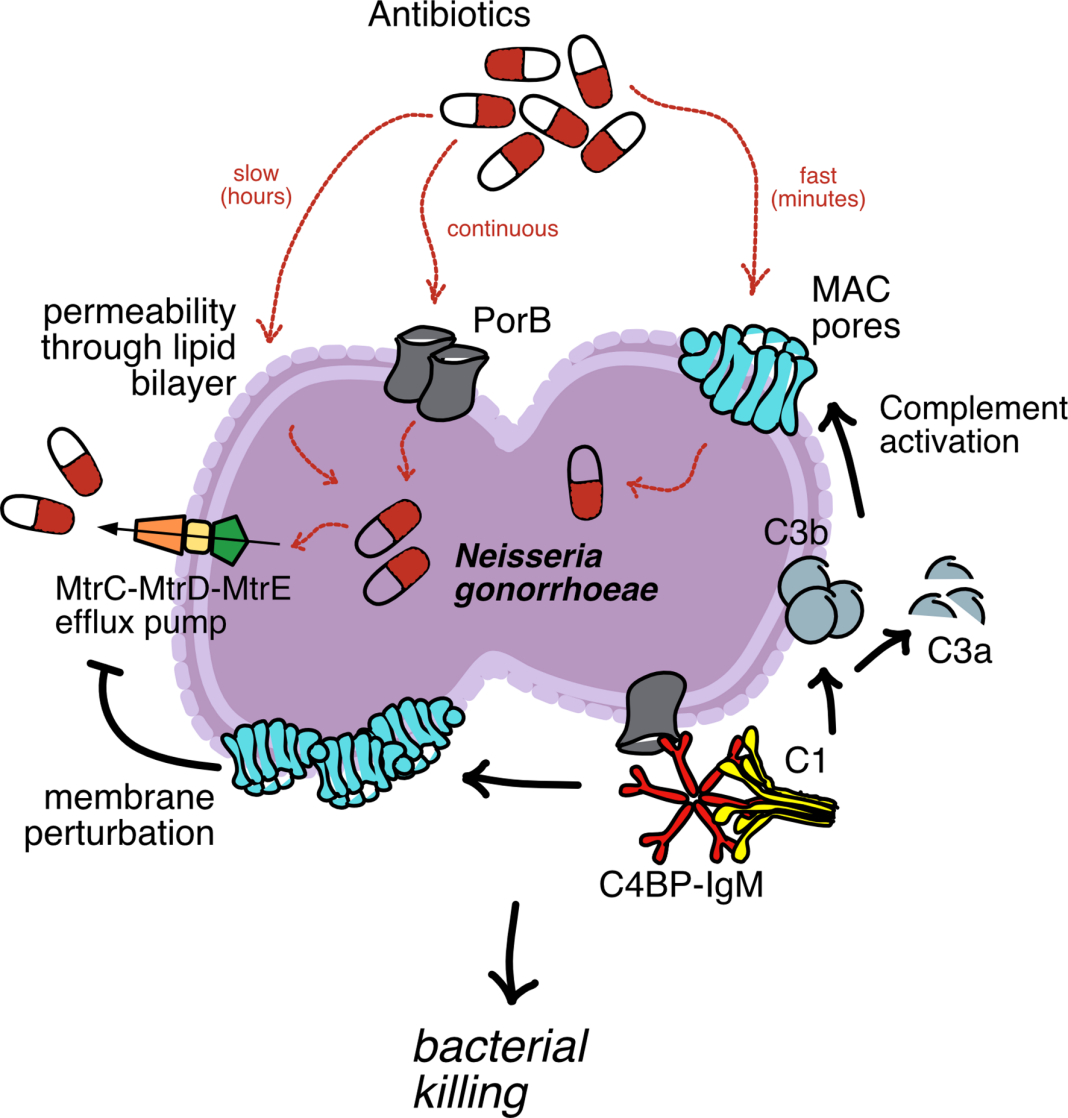
Antibiotics reach their intracellular targets *via* passive permeability across the lipid bilayer or through the membrane porin channels used by bacteria to exchange material with the environment. As a resistance mechanism, bacterial cells actively eliminate antibiotics from inside the cells using efflux pumps located on their membranes. The fusion protein C4BP-IgM binds to bacteria and induces rapid complement activation *via* classical pathway (C1 binding), which rapidly promotes C3b deposition, C3a release and formation of membrane attack complex (MAC) pores on the outer membrane. In turn, MAC pores perturbate the integrity of the membrane that inactivate efflux pumps. Further, MAC pores provide additional channels on bacteria membrane for antibiotic uptake in cell. Together, these events enhance bacteria killing.

## Introduction

*Neisseria gonorrhoeae* is an obligate human pathogen that infects mucosal surfaces and causes the sexually transmitted infection gonorrhea (1). In 2016 the World Health Organization (WHO) estimated the global incidence rate of gonorrhea in persons aged between 15 and 49 years to be 86.9 million (2). If untreated, gonorrhea can cause serious complications, including pelvic inflammatory disease, infertility, and ectopic pregnancy in women and blindness in new-born babies (3–5). Gonorrhea also augments the risk of HIV transmission (6).

An effective anti-gonococcal vaccine remains elusive; control measures mainly include prevention, rapid diagnosis and antibiotic treatment. Gonococci have become resistant to almost all currently available antibiotics (7). The extended-spectrum third-generation cephalosporin ceftriaxone, often given in combination therapy with azithromycin, is the last alternative for first-line monotherapy of gonorrhea and recommended by the WHO and other international or national public health organizations (8–12). Resistance and/or decreased susceptibility to ceftriaxone has however been reported worldwide and occasional gonorrhea treatment failures with recommended dual therapy (ceftriaxone plus azithromycin) and especially ceftriaxone monotherapy was verified internationally (13, 14). Drug-resistant *N. gonorrhoeae* is listed as an ‘urgent threat’ in the 2019 USA CDC report (15), while the WHO listed cephalosporin-resistant, fluoroquinolone-resistant *N. gonorrhoeae* as a high priority in 2017 (16). The spread of azithromycin-resistant *N. gonorrhoeae* strains resulted in the US CDC no longer recommending azithromycin against gonorrhea (11).

*N. gonorrhoeae* has an extraordinary propensity to develop antimicrobial resistance (AMR) through several mechanisms, including inactivation by enzymatic cleavage, alterations of antibiotic targets, and enhanced extrusion or reduced uptake of drugs by efflux/influx pumps (17). The multiple transferable resistance (*mtr*) operon in *N. gonorrhoeae* encodes membrane proteins that form an energy-dependent efflux pump system (MtrC-MtrD-MtrE), which removes drugs and hydrophobic agents from inside the cells into the extracellular space (18). Some gonococcal strains acquire resistance to azithromycin by deletions in the *mtrR* promoter region (MtrR is a repressor of *mtr*CDE) or gain-of-function mutations in mosaic *mtr* efflux pump alleles which result in overproduction/overactivation of efflux pump proteins (19, 20). *Mtr* AMR determinants combined with mutations in genes for the penicillin binding protein 2 (PBP2) and Porin (PorB1b) decrease susceptibility to ceftriaxone (21).

To counteract the increasing spread of AMR *N. gonorrhoeae*, alternative therapies have been proposed. Utilizing the capability of *N. gonorrhoeae* to recruit the complement inhibitor C4b-binding protein (C4BP) to evade complement-dependent killing, we previously created a human fusion protein comprising the gonococcal binding domains of C4BP and the Fc backbone of IgM (C4BP-IgM) (22). Binding of C4BP-IgM to gonococci activated complement and led to the assembly of the membrane attack complex (MAC), resulting in bacterial death. C4BP-IgM promoted complement-mediated killing of several clinical gonococcal isolates and efficiently eradicated bacteria in the mouse vaginal colonization model (22).

Evidence of cooperation between complement activation and antibiotics in killing bacteria dates back to the 1980s (23–27), but the precise molecular mechanisms underlying this effect have remained elusive. Prior studies showed that activation of human complement promoted the ability of polymyxin to produce lesions in the outer membrane of gram-negative bacteria (28, 29). Other reports suggested that sensitization by serum of bacteria to antibiotics is independent of active complement, because serum inactivated at 56°C retained activity. Mechanisms for the cooperative activity posited a role for IgG (30) and an undefined diffusible serum factor (31). A recent study demonstrated that MAC insertion in the outer membrane of gram-negative bacteria sensitized them to nisin and vancomycin, antibiotics that otherwise are active only against gram-positive organisms (32), thereby demonstrating a cooperative effect between complement and antimicrobials. In this study we ask whether C4BP-IgM-mediated complement activation could potentiate killing of *N. gonorrhoeae* by antibiotics and restore susceptibility in drug-resistant isolates.

## Materials and Methods

### Bacterial Cultures

FA1090 strain was used as reference laboratory strain for the majority of *in vitro* experiments (33). FL29 and SA94 gonococcal strains were isolated in Brazil 2015-16 and examined by whole genome sequencing. Both have azithromycin MICs of 4 µg/mL, lack of azithromycin resistance-associated 23S rRNA gene mutations, and have mosaics in the *mtrRCDE* operon resulting in an overexpression of the MtrC-MtrD-MtrE efflux pump that causes the resistance to azithromycin (21). All *N. gonorrhoeae* strains/isolates were stored frozen at -80°C in trypticase soy broth containing 20% glycerol.

### Proteins, Antibodies, and Consumables

Chocolate agar plates were prepared from Gonococcal (GC) agar base (Difco, 228950) (34). GC liquid growth media was prepared as reported (35). CellTrace Calcein Violet (CV, C34858), sytox green (S7020), FM4-64X (F34653), L-homopropargylglycine (HPG), AlexaFluor488 azide (A10266), Click-iT reaction cocktail (C10269), BCA protein assay kit (23225) and Halt Protease and Phosphatase inhibitor (1861281) were obtained from Thermo Fisher Scientific. Hanks Balanced Salt Solution containing 0.15 mM CaCl_2_ and 1 mM MgCl_2_ (HBSS^++^) was purchased from Gibco (14025092). Azithromycin dihydrate (PZ0007), ceftriaxone sodium (PHR1382), ciprofloxacin (17850), cefixime trihydrate (18588), spectinomycin (S4014), anti-human IgM CF647 antibody (SAB4600436), efflux pump inhibitor carbonyl cyanide 3-chlorophenylhydrazone (CCCP, C2759) and fluorescent mounting medium without 4′,6-diamidino-2-phenylindole (DAPI; Dako, S3023) or with DAPI (DUO82040) were purchased from Sigma-Aldrich. *Ornithodoros moubata* complement inhibitor (OmCI) was prepared as described previously (36). C4BP-IgM was constructed by subcloning CCP1–2 domains of C4BP and the CH2–CH4 constant portion of human IgM into the expression vector pClaire. Recombinant C4BP-IgM was stably expressed in adherent CHO cells and purified by affinity chromatography using a specific anti-C4BP antibody as described previously (22). Purified C4BP and C4BP-IgM were fluorescent labeled using AlexaFluor-647 (A30009) or AlexaFluor-488 (A10235) labeling kits from Molecular Probes. Cipro-NBD and Roxi-NBD were synthesized as previously described (37, 38). Human antimicrobial peptide LL-37 (LLGDFFRKSKEKIGKEFKRIVQRIKDFLRNLVPRTES) was synthesized by using Fmoc solid-phase peptide synthesis and purchased from GenScript (Piscataway, NJ, USA).

### Human Complement

Normal Human Serum (NHS) was obtained from whole blood collected from normal healthy adult volunteers (2019/14) as described (22) and stored at -80°C until use. Heat-inactivated serum (HINHS) was prepared by incubation for 30 min at 56°C. C3 and C5 activation were blocked by treating NHS for 30 min on ice with the C3-targeted complement inhibitor Cp40 (50 μM) (39) or with the C5-inhibitor OmCI (25.3 μg/mL), respectively.

### Minimum Inhibitory Concentration (MIC) Evaluation

Gonococci were grown on GC agar plate overnight at 37°C in a 5% CO_2_-enriched atmosphere, and then subcultured in GC liquid medium from OD_600nm_ 0.1 until 0.3-0.4 for 4-5 hours at 37°C, on a rotary shaker. After incubation, bacterial concentration was adjusted to OD_600nm_ 0.1 in new GC liquid medium supplemented with 10% NHS and increasing concentrations of antibiotics (spectinomycin, ciprofloxacin and azithromycin). Bacterial growth was monitored by OD_600nm_ measure every 10 min by Cytation5 reader (BioTek Instruments, Inc).

### Serum Bactericidal Assay

Bactericidal assays with NHS in the presence of C4BP-IgM fusion protein were performed as described previously (22). Bacteria were cultured on GC agar plate overnight and subcultured on fresh plates for at least 5 hours at 37°C in a 5% CO_2_-enriched atmosphere. Two x 10^5^ CFU of harvested *N. gonorrhoeae* diluted in GVB^++^ (5 mM veronal buffer [pH 7.3], 140 mM NaCl, 0.1% gelatin, 1 mM MgCl_2_, and 5 mM CaCl_2_) were incubated with NHS in the presence or the absence of antibiotics and C4BP-IgM fusion protein in a final volume of 100 μL. Concentrations of each component are indicated for each graph. Aliquots of 25 μL of the reaction mixtures were serially diluted in PBS and 10 μL of each dilution were seeded on GC plates in triplicates at the beginning of the assay (input) and after incubation at 37°C for 1, 2 or 3 hours.

### Membrane Perturbation Assays: Sytox Green Staining

Gonococci (FA1090 strain) were grown on GC agar plate overnight at 37°C with 5% CO_2_-enriched atmosphere, and then subcultured in GC liquid medium from OD_600nm_ 0.1 until 0.3-0.4 for 4-5 hours at 37°C shaking. Then, OD_600nm_ was adjusted to 0.1 in new GC liquid medium and supplemented with NHS+/-OmCI (25.3 µg/mL pre-treatment in undiluted NHS for 30 min on ice) and/or C4BP-IgM and/or antibiotics (concentrations indicated for each experiment), all diluted in GVB^++^. Bacteria were then incubated for 3 hours at 37°C with orbital shaking. Then, bacteria were harvested by centrifugation at 5000xg, for 5 min, washed once and incubated for 30 min at RT with 1 μM of sytox green diluted in HBSS^++^, protected from the light. Internalized fluorescent signal was measured after washing bacteria once in HBSS^++^ using Cytation5 reader: Excitation 488/Emission 525+/-20.

### Internalization of Fluorescent Antibiotics by Bacterial Cells

Gonococci (strain FA1090 or FL29) were grown on GC agar plate overnight at 37°C in a 5% CO_2_-enriched atmosphere. Bacteria were then subcultured on a new GC agar plate and incubated for 5 hours at 37°C in a 5% CO_2_-enriched atmosphere. The culture was then harvested and re-suspended in HBSS^++^ to an OD_600nm_ of 2. Some bacteria were treated with 50 μM of efflux pump inhibitor (CCCP) for 30 min at RT. Bacteria were incubated with 20 μM of Cipro-NBD or Roxi-NBD in the presence of 10% NHS+/-OmCI (25.3 μg/mL pre-treatment in undiluted NHS for 30 min on ice) supplemented with 5, 2.5 or 1.25 μg/mL of C4BP-IgM diluted in GVB^++^. Influx of fluorescent antibiotics in bacterial cells was measured by flow cytometry after 15, 30 and 60 min of incubation at 37°C and one wash in HBSS^++^. In parallel, each sample was serially diluted in PBS and 10 μL of each dilution were seeded on GC plates to estimate the survival of bacteria as CFU/mL after overnight growth at 37°C in a 5% CO_2_-enriched atmosphere. For confocal imaging, bacteria were incubated for 30 or 60 min with Cipro-NBD, and then stained for 5 min on ice with 5 μg/mL of FM4-64X diluted in HBSS^++^. After one wash in HBSS^++^, bacteria were spread onto a cover slip, dried and mounted using glass mounting media. LCM800 confocal microscope (Zeiss) with Airyscan mode was used to image the cells.

### Protein Synthesis Labeling *via* Click-iT Chemistry

FA1090 strain was grown on GC agar plate overnight at 37°C in a 5% CO_2_-enriched atmosphere. Bacteria were then subcultured in GC liquid medium from OD_600nm_ 0.1 until OD_600nm_ 0.3-0.4 for about 4 hours at 37°C on a rotary platform shaker. After washing in HBSS^++^ at 37°C, bacteria were adjusted to OD_600nm_ 0.3 in fresh GC liquid media supplemented with 100 μg/mL HPG and 5% NHS, with or without azithromycin (0.25 or 0.05 μg/mL) and 5 μg/mL C4BP-IgM. After 4 hours at 37°C, bacteria were harvested by centrifugation, fixed in 90% methanol during centrifugation at 5000 xg, for 15 min at 4°C, then washed once in HBSS^++^ and permeabilized in 0.5% Triton X-100/HBSS^++^ for 30 min at RT. After centrifugation at 13000 xg, for 3 min, bacterial pellet was washed twice in 1% BSA/HBSS^++^ and stained with 10 μM AlexaFluor488 azide for 30 min at RT using copper-dependent Click-iT reaction cocktail. After washes in 1% BSA/HBSS^++^, bacteria were analyzed by flow cytometry. In parallel, total protein content from bacterial cells was isolated on ice using RIPA buffer (50 mM Tris pH 7.4, 150 mM NaCl, 1% NP40, 0.5% sodium deoxycholate) supplemented with protease inhibitors, and quantified with BCA method. Proteins (10 μg) were loaded on SDS-PAGE gel for detection of fluorescently labeled and silver stained proteins.

### Statistics

Statistical analyzes were performed using GraphPad Prism v8.0. Two-way ANOVA test with Sidak´s multiple comparison was used to analyze the differences between samples in OD growth curve considering various concentrations of the antibiotics or in sytox green uptake considering various concentration of NHS or C4BP-IgM. Two-way ANOVA test with Tukey´s multiple comparison was used to analyze the differences between samples in serum bactericidal assays or antibiotic internalization assays considering two or three time points, respectively. One-way ANOVA test was performed to analyze differences between samples from the reference (NHS) in complement deposition, membrane perturbation and Click-iT protein inhibition assays. Two-way ANOVA test with Dunnett´s multiple comparison was used to analyze differences between samples from reference in serum bactericidal assays with NHS, C4BP-IgM and antibiotics. Significant differences are indicated with asterisks *p < 0.05, **p < 0.01, ***p < 0.005, ****p < 0.0001.

## Results

### Normal Human Serum Reduces the MIC of Antibiotics

**Figure 1:**
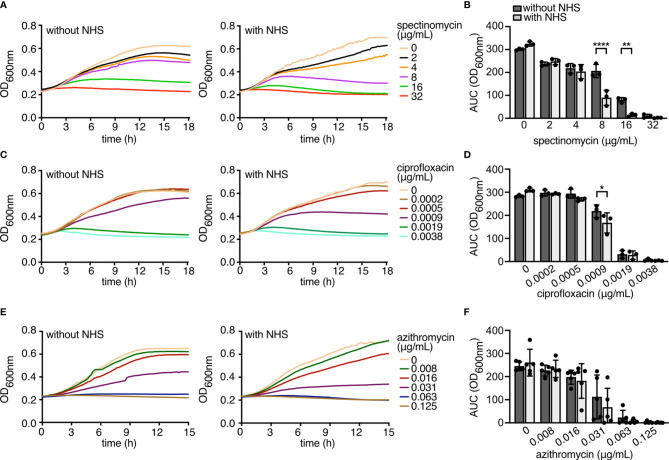
NHS potentiates antibiotic killing of *N. gonorrhoeae* FA1090. **(A, C, E)** FA1090 gonococci were incubated at 37°C with orbital shaking overnight +/- 10% NHS and increasing concentrations of antibiotics: spectinomycin **(A)**, ciprofloxacin **(C)** and azithromycin **(E)**; growth of bacteria was monitored by measuring OD_600nm_ every 10 min. **(B, D, F)** Area under the curve (AUC) of OD_600nm_ were calculated for each concentration of antibiotics. Bars display mean+/-SD, with circles indicating each of 3 **(A–D)** or 5 **(E, F)** independent repeats. Differences among samples were compared using two-way ANOVA with Sidak’s multiple-comparison test. *p < 0.05, **p < 0.01, ****p < 0.0001.

We previously showed that *N. gonorrhoeae* FA1090 resists complement-mediated lysis in 10% NHS because it binds to the human complement inhibitor C4BP (22, 40). When grown overnight in gonococcal liquid medium, FA1090 showed the following MICs: spectinomycin, 16 μg/mL; ciprofloxacin, 0.0019 μg/mL; and azithromycin 0.063 μg/mL. The addition of 10% NHS together with antibiotics to FA1090 cultures significantly reduced OD_600nm_ growth at concentrations equivalent or lower than MIC values for spectinomycin (8 μg/mL *p*<0.0001; 16 μg/mL *p*=0.0011) and ciprofloxacin (0.0009 μg/mL *p*=0.0143), but not significantly for azithromycin. These observations suggested that complement could hamper the growth of gonococci at or just below the MIC of select antibiotics (**Figures 1A–F**).

### Complement Activation by C4BP-IgM Potentiates Killing of *N. gonorrhoeae* by Antibiotics

**Figure 2:**
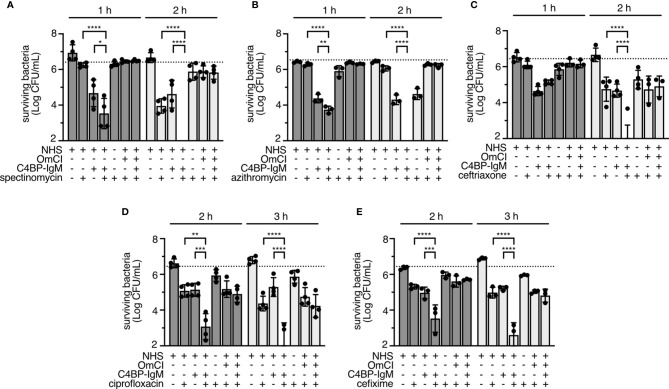
NHS and C4BP-IgM potentiates antibiotic killing of *N. gonorrhoeae* FA1090. **(A–E)** FA1090 gonococci were incubated for 1, 2 or 3 hours with 10% NHS+/-OmCI supplemented with antibiotics (spectinomycin 32 μg/mL, azithromycin 250 ng/mL, ceftriaxone 50 ng/mL, ciprofloxacin 3.75 ng/mL, cefixime 12.5 ng/mL) in the presence or in the absence of 5 μg/mL of C4BP-IgM. Differences among samples were compared using two-way ANOVA with Tukey´s multiple comparison test. Bars display mean+/-SD, with circles indicating 4 **(A, C, D)** or 3 **(B, E)** independent repeats. Horizontal dotted line indicates the starting CFUs of bacteria used in the assay. *p < 0.05, **p < 0.01, ***p < 0.005, ****p < 0.0001.

Previously, we showed that C4BP-IgM could trigger complement activation on the gonococcal surface and kill bacteria (22). We asked whether complement activation by C4BP-IgM could increase the activity of antibiotics. Initially, different concentrations of C4BP-IgM and antibiotics were tested to determine the lowest concentrations causing significant, but partial killing. C4BP-IgM at concentrations below 5 μg/mL did not significantly affect FA1090 survival in 10% NHS (**Supplementary Figure 1A**). When complement-mediated lysis was blocked by OmCI (a C5 inhibitor that blocks formation of MAC), killing of bacteria was abrogated, confirming the complement-driven bactericidal activity of C4BP-IgM (**Supplementary Figure 1A**). Then, we identified concentrations of antibiotics which resulted in a partial killing of bacteria following incubation with or without serum for 2 h (spectinomycin, azithromycin and ceftriaxone) or 3 h (cefixime and ciprofloxacin). These concentrations were: 32 μg/mL for spectinomycin, 250 ng/mL for azithromycin, 50 ng/mL for ceftriaxone, 3.75 ng/mL for ciprofloxacin and 12.5 ng/mL for cefixime (**Supplementary Figures 1B–F**). Using a C4BP-IgM concentration of 5 μg/mL, we observed full killing of FA1090 bacteria after 2 hours of incubation with serum and spectinomycin, azithromycin and ceftriaxone (**Figures 2A–C**). Significant reductions in survival were also seen with the combinations of NHS, C4BP-IgM and ciprofloxacin or cefixime (**Figures 2D, E**). Blocking complement deposition by heat or with the complement-inhibitor OmCI abrogated killing of bacteria, suggesting that complement activation induced by C4BP-IgM potentiated antibiotic-mediated killing. While bacteria were completely killed following 2 hours of incubation with spectinomycin, azithromycin or ceftriaxone and C4BP-IgM plus NHS, modest survival at this time point was noted with ciprofloxacin and cefixime. Extension of the end-point of assays with ciprofloxacin and cefixime to 3 hours revealed a further drop in colony forming units (CFUs), suggesting slower killing kinetics with the latter antibiotics. Since partial killing was still evident with ciprofloxacin and cefixime even when complement was blocked with OmCI (**Figures 2D, E**), we speculated that components of human serum other than complement, i.e. endogenous antimicrobial peptides, may also have an effect on bacterial killing by antibiotics, as previously published (41). However, addition of the synthetic antimicrobial peptide LL-37 to NHS+C4BP-IgM did not enhance killing of bacteria, suggesting that LL-37 does not potentiate killing of gonococci by active complement (**Supplementary Figure 2**).

### Complement Activation Promoted by C4BP-IgM Induces Membrane Damage of *N. gonorrhoeae*

**Figure 3:**
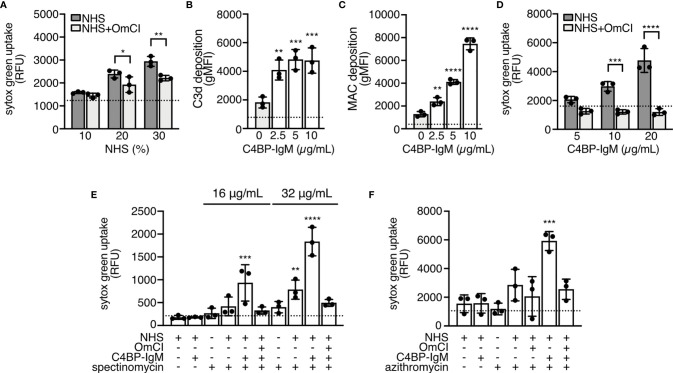
NHS and C4BP-IgM induce membrane damage of *N. gonorrhoeae* FA1090 through MAC formation. **(A, D)** FA1090 gonococci were incubated for 3 hours with increasing concentration of NHS (10%, 20%, 30%) **(A)** or with 10% NHS and increasing concentrations of C4BP-IgM (5, 10 and 20 μg/mL) **(D)**. Internalization of sytox green was measured after 30 min incubation. Differences among samples were compared using two-way ANOVA with Sidak´s multiple comparison test. Dotted line indicates fluorescent value in the absence of NHS or C4BP-IgM, respectively. **(B, C)** FA1090 gonococci were incubated with 10% NHS and 2.5, 5 or 10 μg/mL of C4BP-IgM. Deposition of C3d and MAC was measured by flow cytometry. Dotted line indicates value in the presence of HINHS. One-way ANOVA with Dunnett´s multiple comparison test was performed considering NHS as reference. **(E, F)** FA1090 gonococci were incubated for 3 hours in the presence or in the absence of 10% NHS+/-5 μg/mL C4BP-IgM and 16 or 32 μg/mL of spectinomycin or 125 ng/mL of azithromycin. Internalization of sytox green was measured after 30 min incubation. One-way ANOVA with Dunnett´s multiple comparison test was performed considering NHS as reference. Dotted line indicates fluorescent value in the presence of bacteria alone. In all graphs n = 3. *p < 0.05, **p < 0.01, ***p < 0.005, ****p < 0.0001.

To elucidate how active complement potentiates gonococci killing by antimicrobials we investigated whether perturbation of the bacterial membrane by complement permitted antibiotic penetration. Even though FA1090 was resistant to MAC-mediated lysis in NHS (**Supplementary Figure 3A*****;*** solid grey bars), NHS allowed influx of the cell impermeable reagent sytox green when incubated with increasing concentrations of active serum for 3 hours (**Figure 3A**). Blocking the complement cascade with the C5-inihbitor OmCI limited (but did not eliminate) sytox green entry into bacterial cells suggesting that MAC pores perturbed gonococcal membranes. As described previously (22), C4BP-IgM bound to FA1090 and efficiently promoted complement C3 fragment (as measured by C3d) and MAC deposition on the bacterial surface in a dose-dependent manner (**Figures 3B, C**). Consequently, adding increasing concentrations of C4BP-IgM to serum correlated with increasing sytox uptake by gonococci, which was reduced to baseline levels in the presence of OmCI, further confirming membrane perturbation by MAC (**Figure 3D**). We then explored membrane permeability in the presence of 10% NHS, 5 μg/mL of C4BP-IgM and antibiotics (spectinomycin or azithromycin); the reaction mixtures resulted in only partial bacterial killing. When C4BP-IgM or antibiotics were added singly to NHS they induced minor ingress of the fluorescent dye into FA1090 cells. Interestingly, the presence of C4BP-IgM in NHS together with each antibiotic significantly increased sytox green influx into FA1090 gonococci, indicating substantially greater membrane perturbation (**Figures 3E, F**). Increasing concentrations of spectinomycin added to 10% NHS resulted in accumulation of fluorescence inside bacteria, further indicating that complement activation plays a key role in sensitizing bacteria to antibiotics (**Supplementary Figure 3B**).

### Antibiotic Influx Into Bacterial Cells Is Promoted by Complement Activation and Further Enhanced by C4BP-IgM

**Figure 4:**
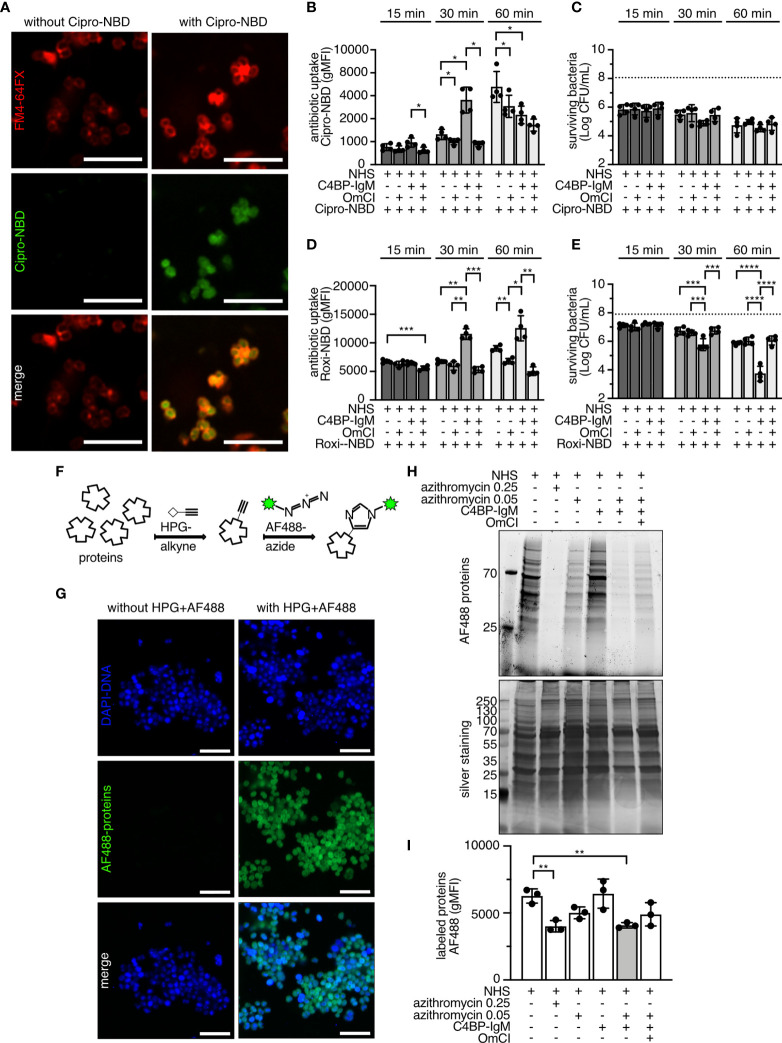
NHS and C4BP-IgM promote influx and intracellular activity of antibiotics in *N. gonorrhoeae*. **(A)** Airyscan confocal fluorescent microscopy of live FA1090 labeled with FM4-64FX membrane dye and treated with or without 20 μM of Cipro-NBD for 60 min at 37°C. Scale bar: 5 μm. **(B–E)** FA1090 gonococci were incubated with 20 μM of Cipro-NBD or Roxi-NBD for 15, 30 and 60 min at 37°C in the presence of 10% NHS+/-OmCI with or without 5 (Cipro-NBD) or 2.5 μg/mL (Roxi-NBD) of C4BP-IgM. Internalized fluorescent antibiotic is measured as fluorescent signal (gMFI). Survival of bacteria is indicated as Log(CFU/mL) with horizontal dotted line referring to average of Log(CFU/mL) of bacteria recovered in the absence of probes. Bars display mean+/-SD with circles indicating independent repeats; n = 4. Two-way ANOVA with Tukey´s multiple comparison was performed to analyze differences among samples. *p < 0.05, **p < 0.01, ***p < 0.005, ****p < 0.0001. **(F)** Schematic representation of AF488-labeling of newly synthesized proteins *via* Click-iT chemistry. **(G)** Airyscan confocal fluorescent microscopy of live FA1090 labeled with DAPI DNA dye after 4 hours incubation with 100 μg/mL HPG-alkyne at 37°C followed by 10 μM AF488-azide *via* Click-iT chemistry. Scale bar: 5 μm. **(H, I)** FA1090 gonococci were incubated for 4 hours at 37°C in the presence of 100 μg/mL HPG-alkyne, 5% NHS+/-5 μg/mL C4BP-IgM and 0.25 or 0.05 μg/mL of azithromycin. Newly synthesized proteins were labeled with 10 μM AF488-azide *via* Click-iT chemistry and detected on SDS-PAGE in fluorescent or by silver staining **(H)** and in flow cytometry **(I)**, n = 3. One-way ANOVA with Dunnett´s multiple comparison test was performed considering NHS as reference. *p < 0.05, **p < 0.01, ***p < 0.005, ****p < 0.0001.

The availability of fluorophore (NBD)-labeled versions of ciprofloxacin and roxithromycin (a macrolide, related to azithromycin) (37, 38) allowed us to study whether complement activation promoted the influx of antibiotics. Cipro-NBD and Roxi-NBD uptake by gonococci in the presence or in the absence of 10% NHS and 5 (Cipro-NBD) or 2.5 μg/mL (Roxi-NBD) of C4BP-IgM, was monitored over time. Cipro-NBD (20 μM) was efficiently internalized by FA1090 after 60 min, as shown in the confocal microscopy images in **Figure 4A** where bacterial membranes were stained with the lipophilic probe FM4-64FX. NHS increased both Cipro-NBD and Roxi-NBD influx at 60 min, which was significantly decreased when the C5 was blocked with OmCI (**Figures 4B, D**). Interestingly, the presence of C4BP-IgM, as enhancer of complement activation in NHS, augmented the amount and accelerated the internalization of both probes in FA1090 cells as early as 30 min of incubation. Uptake of the fluorescent antibiotics (which retain antimicrobial activity) resulted in the killing of bacteria over time (**Figures 4C, E**), which was significantly enhanced when serum plus C4BP-IgM was supplemented with Roxi-NBD (**Figure 4E**). Taken together, these data suggest that the fusion protein boosts complement-mediated membrane permeabilization, which promotes antibiotic influx into bacterial cells resulting in cell death.

### Antibiotic Activity Inside Bacterial Cells Increases When Complement Is Activated by C4BP-IgM

Another method to verify the internalization of macrolides into the cells is to evaluate their effect on protein synthesis. Azithromycin is a ring-expanded macrolide derivative, which permeates into the cell interior through the lipid bilayer. Once inside the bacterium, azithromycin interacts with the 50S ribosomal subunit and inhibits protein synthesis, which in turn arrests bacterial growth. To monitor protein synthesis and azithromycin inhibitory activity, we used the Click-IT technology based on the incorporation of homopropargylglycine (HPG), an amino acid analog of methionine containing an alkyne moiety, which was then detected by the AF488 conjugated-azide through direct coupling (**Figure 4F**). After 4 hours of incubation with the modified amino acid, the majority of the proteins present inside bacterial cells became fluorescently labeled, as a result of incorporation of the exogenous amino acid supplemented in the medium (**Figure 4G**). Protein synthesis was not significantly altered in the presence of NHS with or without C4BP-IgM at sub-bactericidal concentrations, or in the presence of concentrations of azithromycin (0.05 μg/mL) in NHS that caused only partial killing (**Figures 4H, I**). Interestingly, when azithromycin (0.05 μg/mL) was added to serum containing C4BP-IgM, the quantity of fluorescent proteins significantly decreased indicating that the inhibitory activity of azithromycin on protein synthesis increased when complement activation was boosted by C4BP-IgM. It is worth noting that a 5-fold higher concentration of azithromycin (0.25 μg/mL) was required to obtain an equivalent level of inhibition of protein synthesis seen in the presence of C4BP-IgM. Additionally, adding OmCI to the mixture with NHS+C4BP-IgM and azithromycin 0.05 μg/mL partially restored protein synthesis to levels observed with azithromycin (0.05 μg/mL) in NHS, further confirming the impact of complement-mediated MAC pores in increasing the activity of azithromycin.

### Complement Activation Through C4BP-IgM Restores Antibiotic Susceptibility of Two Azithromycin-Resistant *N. gonorrhoeae* Strains

**Figure 5:**
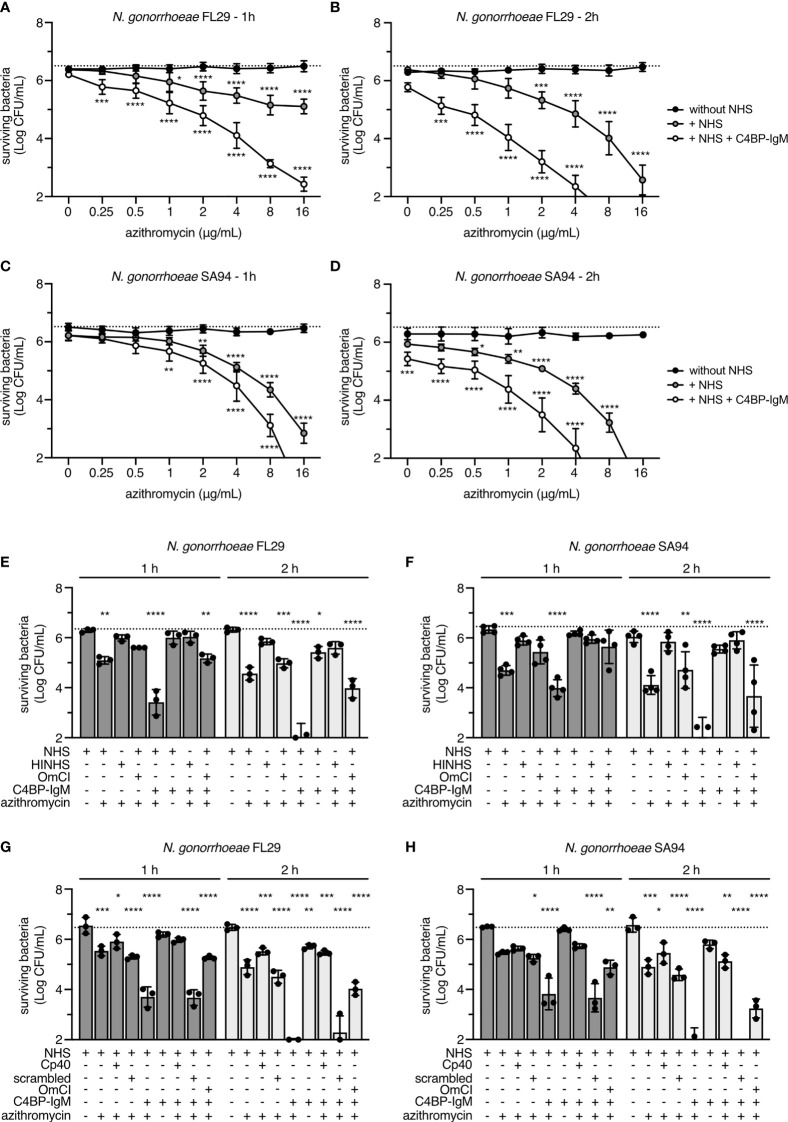
Complement activation in NHS cooperates with antibiotics in killing two azithromycin-resistant strains of *N. gonorrhoeae*. FL29 **(A, B)** and SA94 **(C, D)** gonococci were incubated for 1 and 2 hours with increasing concentrations of azithromycin in the presence or in the absence of 10% NHS and 0.6 μg/mL or 0.16 μg/mL of C4BP-IgM, respectively. At each azithromycin concentration, differences from samples without NHS were compared using two-way ANOVA with Dunnett´s multiple comparison test. FL29 **(E, G)** and SA94 **(F, H)** gonococci were incubated for 1 or 2 hours with 10% NHS, HINHS or NHS+OmCI or NHS+Cp40 (50 μM) supplemented with 4 μg/mL of azithromycin, in the presence or in the absence of 0.6 μg/mL or 0.16 μg/mL of C4BP-IgM, respectively. Scrambled peptide is used as irrelevant protein for Cp40-treated samples. Two-way ANOVA with Dunnett´s multiple comparison test was performed considering NHS as reference. Bars display mean+/-SD, with circles indicating 4 **(A–D, F)** and 3 **(E, G, H)** independent repeats. In all graphs horizontal dotted line refers to the starting number of bacteria used in the assay. *p < 0.05, **p < 0.01, ***p < 0.005, ****p < 0.0001.

Encouraged by the results obtained with the laboratory strain FA1090, we then explored the activity of complement in combination with antibiotics on two selected clinical isolates (FL29 and SA94) of *N. gonorrhoeae*, which are resistant to azithromycin (MIC by Etest = 4 µg/mL) (23). These clinical isolates were chosen because their mechanism of azithromycin resistance was overexpression of the MtrC-MtrD-MtrE efflux pump caused by a mosaic *mtrRCDE* operon that we hypothesized could be overcome by enhanced macrolide entry facilitated by MAC. Firstly, we verified that C4BP-IgM bound to both strains and promoted complement-mediated killing (**Supplementary Figures 4A–C**). Concentrations of C4BP-IgM that resulted in only partial killing for each strain were identified to be 0.6 μg/mL for FL29 and 0.16 μg/mL for SA94. Both strains survived completely when incubated for 2 hours with concentrations of azithromycin up to 16 μg/mL (**Figures 5A–D**). The presence of NHS significantly decreased survival of bacteria in the presence of azithromycin above 1 μg/mL. Notably, a further significant reduction in survival for both strains was observed with supplementation of C4BP-IgM at all concentrations of azithromycin tested in a time dependent manner. In a parallel control reaction, azithromycin at 16 μg/mL completely killed strain FA1090. Taken together, these results suggest an additive effect between C4BP-IgM and azithromycin in killing gonococci.

To verify the contribution of complement activation in augmenting killing by 4 μg/mL azithromycin, we then tested the effect of C4BP-IgM in serum that was inactivated by heating at 56°C or with OmCI. Interestingly, while heat inactivation of human serum completely rescued bacteria survival both in the presence or in the absence of the fusion protein, complement inhibition by OmCI only partially prevented bacterial killing induced by C4BP-IgM, suggesting the presence of additional heat-labile bactericidal components in serum that potentiate azithromycin activity on gonococci (**Figures 5E, F**). C3a generated by complement activation also shows bactericidal activity (42). To test whether C3 fragments generated during complement activation by C4BP-IgM facilitated killing by azithromycin in killing gonococci, we used the selective C3 inhibitor compstatin (Cp40) (39). Blocking C3 cleavage with 50 μM of Cp40 abrogated C4BP-IgM-mediated killing (**Supplementary Figure 4B**), and protected gonococci from killing by the combination of serum and azithromycin to a significantly greater extent than OmCI (**Figures 5G, H**). These results indicate that C4BP-IgM mediated complement activation through the level of C3 activation may also sensitize bacteria to macrolides.

### C4BP-IgM Promotes Antibiotic Entry Into the Cell of Azithromycin-Resistant *N. gonorrhoeae* Strain

**Figure 6:**
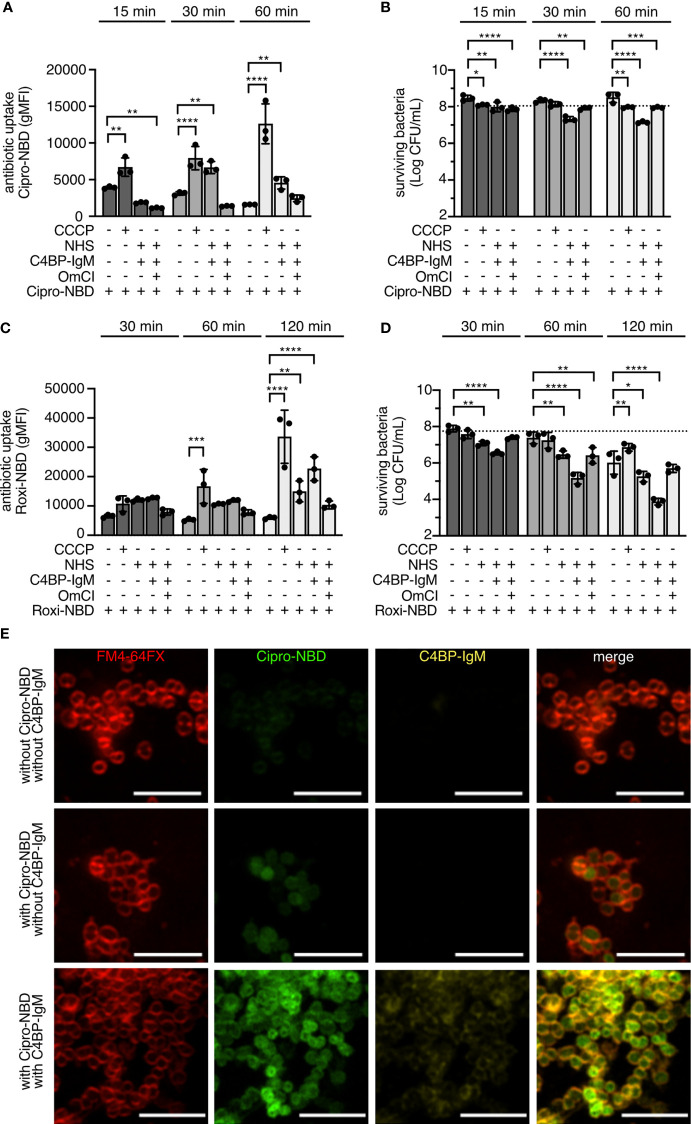
NHS and C4BP-IgM promote internalization of antibiotics in *N. gonorrhoeae* FL29. FL29 gonococci were incubated with 20 μM of Cipro-NBD for 15, 30 and 60 min **(A, B)** or 20 μM of Roxi-NBD for 30, 60 and 120 min **(C, D)** at 37°C in the presence of 10% NHS+/-OmCI with or without 50 μM of CCCP or 1.25 μg/mL of C4BP-IgM. Internalized fluorescent antibiotic is measured as fluorescent signal (gMFI). Survival of bacteria is indicated as Log(CFU/mL), and horizontal dotted line indicates the average of Log(CFU/mL) of bacteria recovered in the absence of probes. Bars display mean+/-SD with circles indicating independent repeats; n = 3. Two-way ANOVA with Dunnett´s multiple comparison test was performed to analyze differences of samples in comparison to bacteria+probe. *p < 0.05, **p < 0.01, ***p < 0.005, ****p < 0.0001. **(E)** Airyscan confocal fluorescent microscopy of live FL29 labeled with FM4-64FX membrane dye and treated with or without 20 μM of Cipro-NBD for 30 min at 37°C in the presence or in the absence of NHS and C4BP-IgM, the latter detected using an Alexa Fluor-647 labeled anti-human IgM. Scale bar: 5 μm.

To show that active complement mediates internalization of antibiotics in gonococci, uptake of NBD-labeled antibiotics was monitored in the azithromycin-resistant strain FL29. Treatment of bacteria with the efflux pump inhibitor CCCP, which disrupts the proton gradient driving efflux pumps (overactivation of the MtrC-MtrD-MtrE efflux pump in FL29 causes the macrolide resistance and increases its MIC to ciprofloxacin), resulted in a significant influx of both Cipro-NBD and Roxi-NBD in a time dependent manner (**Figures 6A, C**). However, survival of bacteria was barely affected, which might suggest that inhibition was not stably maintained over time (**Figures 6B, D**). The presence of NHS together with C4BP-IgM significantly augmented internalization of the antibiotic-NBD probes overtime. Consequently, survival of bacteria was significantly hampered when incubated with both fluorescent antibiotics and NHS supplemented with the fusion protein (**Figures 6B, D**). Complement inhibition by OmCI completely abrogated influx of both fluorescent antibiotics into gonococci, resulting in survival of bacteria. Confocal images of live FL29 gonococci treated with Cipro-NBD in the absence of NHS for 30 min revealed that only few bacteria contained the fluorescent probe, providing evidence that most cells efficiently removed the drug by efflux pumps that are overexpressed in this strain. Of note, when bacterial cells were coated with C4BP-IgM, almost all cells were stained with the fluorescent green probe, suggesting that the fusion protein helped overcome the hyperactive efflux pumps and promoted drug influx (**Figure 6E**).

## Discussion

Antimicrobial resistance has emerged worldwide in many pathogenic bacteria, including *N. gonorrhoeae*. The recent treatment failures of ceftriaxone, either as monotherapy or even in combination with azithromycin highlight the urgent need to develop alternative therapeutic approaches against gonorrhea (7, 15). We previously showed the efficacy of C4BP-IgM fusion protein in eliminating gonococci through complement-mediated lysis (22). In the present study we propose using C4BP-IgM as an antimicrobial adjuvant for the treatment of gonococcal infections. The ability of C4BP-IgM to increase the efficacy of antimicrobials relies on the ability of the fusion protein to boost complement activation on the microbial surface. Complement-induced MAC pores facilitate uptake of antimicrobials, which in turn enhances their intracellular concentration and activity. In particular, our results demonstrate that C4BP-IgM potentiates the activity of azithromycin against two macrolide-resistant strains carrying *mtr* efflux pump mutations. We therefore propose C4BP-IgM as an alternative strategy to bypass the bacterial membrane barrier and restore the activity of antibiotics against resistant strains with hyperactive efflux pumps. We acknowledge that enhanced penetration of antibiotics may not overcome resistance in strains where mutations abrogate the binding of antibiotics to their targets (e.g., mutations in the 23S rRNA of the 50S ribosomal subunit for azithromycin and GyrA for quinolones).

Early vaccine development efforts against *N. gonorrhoeae* were not successful in large part because antigenic variability of candidate vaccine antigens led to strain-specific immunity (43, 44). Effective antimicrobial treatment remains an essential element in the management and control of gonococcal infections. However, the rapid development of AMR in gonococci has repeatedly compromised treatment (17). The combination of antibiotics with membrane permeabilizing agents as adjunctive treatments may be a viable antibacterial approach (45). Such agents increase bacterial membrane fluidity or reduce efflux of drugs. Numerous examples of this approach have been described for gram-negative bacteria, including use of: polymyxins (29), the anti-protozoal drug pentamidine (46), the human α-lactalbumin–oleic acid complex HAMLET (47), and natural or synthetic inhibitors of efflux pumps (48). Interestingly, *ex vivo* assays using human blood showed that human complement activation on the outer membrane of bacteria sensitizes some gram-negative strains to antibiotics otherwise specific for gram-positive bacteria (32). Given its ability to insert MAC pores into the bacterial membrane, we hypothesized that the C4BP-IgM fusion protein would augment antimicrobial activity in a similar way to the aforementioned permeabilizing agents. Indeed, C4BP-IgM disrupts gonococcal membranes in the presence of human active serum and promotes the uptake of antibiotics. Intracellular accumulation of the drugs resulted in enhanced antimicrobial activity that accelerated gonococcal killing. Rapid and complete killing limits the opportunity for resistant organisms to emerge. Several cell permeabilizing agents also act on eukaryotic cells, which could result in host toxicity. C4BP-IgM has an advantage because it neither binds to nor enhances complement deposition on human cells, as we showed previously (22). Other molecules that enhance complement activation on bacteria may also enhance activity of antimicrobials. As an example, fusion proteins which combine bacterial binding domains of FH with constant portions of immunoglobulins (49, 50) could also function as membrane permeabilizers in a manner similar to C4BP-IgM.

Factors in serum other than complement may contribute to killing of some gram-negative bacteria independently of MAC (51), therefore we cannot exclude the cooperation of other antibacterial peptides in NHS in killing gonococci by ciprofloxacin and azithromycin. Interestingly, we observed that C3 fragments generated by C4BP-IgM in serum cooperates with antibiotics to kill gonococci. A previous study showed that C3a possesses bactericidal properties (42). While blocking MAC formation with the C5 inhibitor OmCI only partially rescued killing of gonococci by the combination of azithromycin, NHS and C4BP-IgM, heat inactivation of NHS (blocks C3 deposition) or the addition of compstatin (blocks C3 cleavage) to NHS abrogated killing. These data suggest that antimicrobial activities upstream in the complement cascade – likely C3 activation – may also augment killing by antibiotics.

C4BP-IgM triggers rapid complement activation, which is essential to form MAC pores. A recent study revealed that MAC pores are flexible toroid-shaped holes with an inner diameter of about 30 Å (3 nm) that adopt either an open or a closed conformation (52). Upon complement activation, the MAC pores may provide additional channels for antibiotics to reach their targets in the intracellular compartments of bacteria. Thus, molecular mechanisms that reduce resistance to antibiotics are likely the result of a dual effect on membrane disruption: more antibiotic entry through MAC pores and less drug efflux because of reduced efflux pump activity.

The efflux pump complex expressed by the *mtrCDE* operon of *N. gonorrhoeae* expels various hydrophobic agents including antibiotics and antibacterial peptides from cells. Gonococci possess transcriptional control systems to modulate expression of their efflux pumps (53). This could result in increased bacterial proliferation before effective antibiotic therapy can be initiated when overexpression of *mtr* genes protects them from these agents. Reducing the dosage of the antibiotics using adjunctives such as C4BP-IgM could limit the selection of mutants overexpressing MtrC-MtrD-MtrE efflux pumps or the production of otherwise silent MDR transporters. Combination treatment also may limit the side effects of single agents that would otherwise be used in larger doses, while potentiating the antimicrobial attack towards different targets. Interestingly, despite the fact that some AMR determinants may increase bacterial survival and fitness during infection (54), overexpression of the MtrC-MtrD-MtrE efflux pump can result in a fitness cost during cervical colonization (55).

A limitation of our approach is that C4BP-IgM as ‘permeabilizing agent’ for antibiotics would work only on those bacteria that are able to bind C4BP, which is about 45% of surveyed strains (22, 40). Nevertheless, our results provide proof-of-principle that triggering complement activation can restore susceptibility of gonococci with overactive MtrC-MtrD-MtrE efflux pumps to macrolides.

In this study we demonstrated the adjuvant activity of C4BP-IgM fusion protein for certain antibiotics previously or currently used to treat gonorrhea. Therefore, C4BP-IgM could be considered for topical administration at the site of infection (for example, intravaginally) in conjunction with systemically administered antibiotics.

## Conclusion

In conclusion, our data provide proof-of-principle to enhance complement activation on microbes, which in turn potentiates antibiotic efficacy and restores susceptibility to select resistant strains. This approach offers an innovative option to combat the global problem of antimicrobial resistance.

## Data Availability Statement

The raw data supporting the conclusions of this article will be made available by the authors, without undue reservation.

## Ethics Statement

The studies involving human participants were reviewed and approved by the ethical committee in Lund (Permit 2019/14). The patients/participants provided their written informed consent to participate in this study.

## Author Contributions

SB, KM, MU, SR, and AB designed research studies and analyzed data. SB and KM conducted experiments. MS and MAB supplied fluorescent probes and gave technical advice. MU and MLB provided strains and data for gonococcal clinical isolates. JP provided the purified LL-37. SB, SR, and AB wrote the manuscript. All authors contributed to the article and approved the submitted version.

## Funding

The study was supported by grants from Swedish Research Council (2018-02392), Torsten Söderberg Foundation (MT3/18), The Österlund Foundation (to AB) as well as grants from Sten K. Johnsons Foundation (2019), the Tore Nilson’s Foundation (2019-00750), the Royal Physiographic Society of Lund (40824), the O. E. och Edla Johanssons Foundation (2020 and 2021), The Lars Hierta Memorial Foundation (FO2020-0257), Längmanska kulturfonden (BA20-1272, BA21-0550) and Clas Groschinskys Fondation (M21106) (to SB). MS was supported by an Australian Postgraduate Award and an Institute for Molecular Biosciences Research Advancement Award. SR was supported by National Institutes of Health grants R01 AI32296 and R44 AI147930.

## Conflict of Interest

The authors declare that the research was conducted in the absence of any commercial or financial relationships that could be construed as a potential conflict of interest.

## Publisher’s Note

All claims expressed in this article are solely those of the authors and do not necessarily represent those of their affiliated organizations, or those of the publisher, the editors and the reviewers. Any product that may be evaluated in this article, or claim that may be made by its manufacturer, is not guaranteed or endorsed by the publisher.

## Supplementary Material

Supplementary Figure 1 Subtotal concentrations of C4BP-IgM and antibiotics on *N. gonorrhoeae*. FA1090 gonococci were incubated with increasing concentrations of C4BP-IgM **(A)** or of spectinomycin, azithromycin, ceftriaxone, ciprofloxacin and cefixime **(B–F)** with or without 10% NHS for time indicated for each graph. Circles indicate mean+/-SD of independent repeats; n = 3. Comparisons between NHS alone and NHS plus C4BP-IgM, or antibiotic alone and NHS plus antibiotic at the respective time point were made by two-way ANOVA with Dunnett´s multiple comparison test. In all graphs survival of bacteria was analyzed as Log(CFU/mL) and horizontal dotted line refers to the starting number of bacteria used in the assay. *p < 0.05, **p < 0.01, ***p < 0.005, ****p < 0.0001.

Supplementary Figure 2 Effect of LL-37 on bactericidal activity of serum against *N. gonorrhoeae.* FA1090 gonococci were incubated for 2 or 3 hours with 10% of NHS+/-OmCI in the presence or in the absence of 0.8 μg/mL of LL-37 with 20 μg/mL of C4BP-IgM. Two-way ANOVA with Tukey´s multiple-comparison test was performed to analyze differences among samples within the same hour. Bars indicate mean+/-SD, and circles refer to 3 independent repeats. Survival of bacteria was analyzed as Log(CFU/mL) and horizontal dotted line indicates the starting number of bacteria used in the assay. *p < 0.05, **p < 0.01, ***p < 0.005, ****p < 0.0001.

Supplementary Figure 3 FA1090 susceptibility to NHS or spectinomycin. **(A)** FA1090 gonococci were incubated for 30 min at 37°C with increasing concentrations of NHS, and survival was estimated as Log(CFU/mL) with horizontal dotted line indicates the starting number of bacteria used in the assay. Sample with 10% NHS + C4BP-IgM (10 μg/mL) is used as a positive control for bactericidal activity of serum. **(B)** FA1090 gonococci were incubated for 3 hours with or without 10% NHS and increasing concentrations of spectinomycin. Internalization of sytox green was measured after 30 min incubation. In all graphs, bars indicate mean+/-SD, and circles show 3 independent repeats.

Supplementary Figure 4 Azithromycin resistant strains of *N. gonorrhoeae*. **(A)** Flow cytometric determination of binding of fluorescently labeled C4BP and C4BP-IgM to FL29 and SA94 gonococci after 30 min of incubation at 37°C. FA1090 and F62 were used as controls for positive and negative binding, respectively.**(B, C)** FL29 and SA94 gonococci were incubated for 1 or 2 hours with increasing concentrations of C4BP-IgM in the presence of 10% NHS+/-OmCI. Circles display mean+/-SD of 3 independent experiments. At each time point, differences between samples with C4BP-IgM and those without the protein were compared using two-way ANOVA with Dunnett´s multiple comparisons test. **(D)** FL29 gonococci were incubated for 1 or 2 hours with 10% NHS+/-OmCI or increasing concentrations of Cp40 or scrambled peptide (10, 50 or 100 μM) in the presence or in the absence of 2.5 μg/mL of C4BP-IgM. Scrambled peptide is used as irrelevant protein for Cp40-treated samples. Two-way ANOVA with Dunnett´s multiple comparisons tests was performed considering sample with NHS alone as reference. Bars display mean+/-SD with circles indicating independent repeats; n = 3. Horizontal dotted line indicates the starting number of bacteria used in the assay. *p < 0.05, **p < 0.01, ***p < 0.005, ****p < 0.0001.

## Abbreviations

AF, Alexa Fluor; GC, gonococcal; BSA, bovine serum albumin; C4BP, C4-binding protein; CCCP, efflux pump inhibitor carbonyl cyanide 3-chlorophenylhydrazone; CFU, colony forming unit; DAPI, diamidino-2-phenylindole; gMFI, geometric mean fluorescence intensity; GVB, gelatin veronal buffer; Ig, immunoglobulin; HBSS, Hanks Balanced Salt Solution; MAC, membrane attack complex; NHS, normal human serum; OD, optical density; OmCI, *Ornithodoros moubata* complement inhibitor; AMR, antimicrobial resistance; Cp40, compstatin.
