# Supplementary figures and images for: Serum Complement Activation by C4BP-IgM Fusion Protein Can Restore Susceptibility to Antibiotics in Neisseria gonorrhoeae
Source: Front Immunol. 2021 Sep 1;12:726801. doi: 10.3389/fimmu.2021.726801 (PMC8440848; doi:10.3389/fimmu.2021.726801)

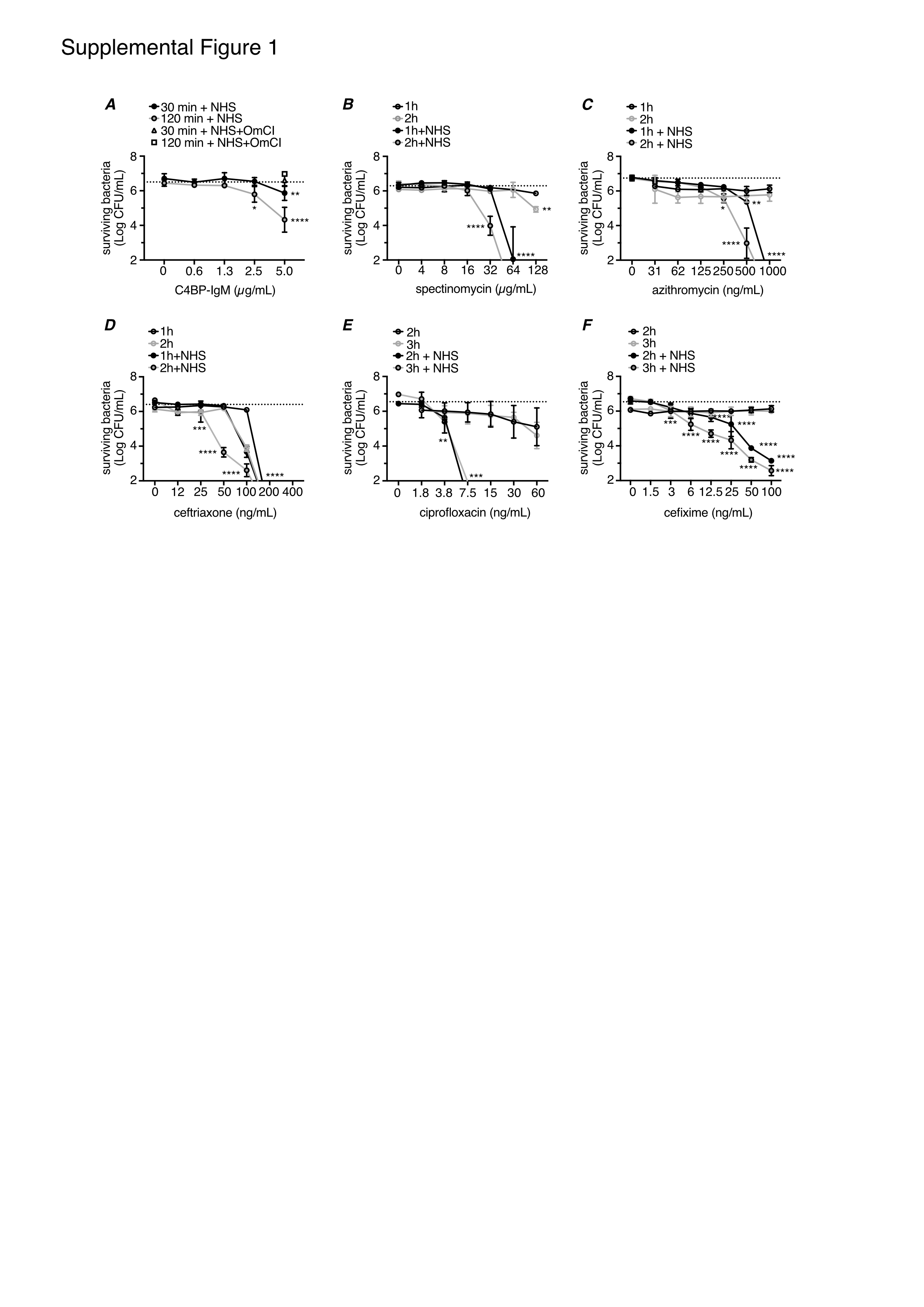

Supplement: Supplementary Figure 1 — Subtotal concentrations of C4BP-IgM and antibiotics on N. gonorrhoeae. FA1090 gonococci were incubated with increasing concentrations of C4BP-IgM (A) or of spectinomycin, azithromycin, ceftriaxone, ciprofloxacin and cefixime (B–F) with or without 10% NHS for time indicated for each graph. Circles indicate mean+/-SD of independent repeats; n = 3. Comparisons between NHS alone and NHS plus C4BP-IgM, or antibiotic alone and NHS plus antibiotic at the respective time point were made by two-way ANOVA with Dunnett´s multiple comparison test. In all graphs survival of bacteria was analyzed as Log(CFU/mL) and horizontal dotted line refers to the starting number of bacteria used in the assay. *p < 0.05, **p < 0.01, ***p < 0.005, ****p < 0.0001. [file Image_1.tiff]

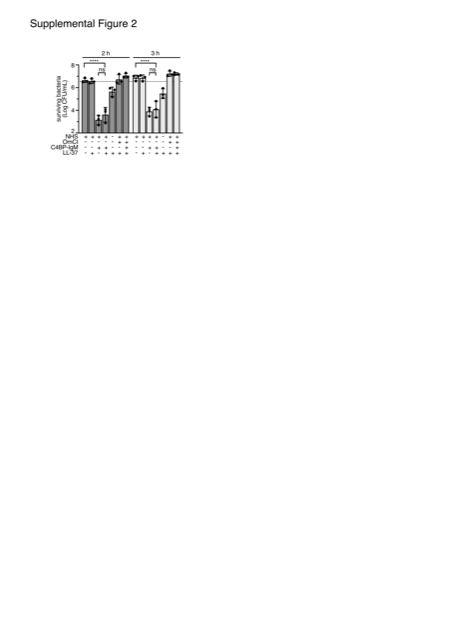

Supplement: Supplementary Figure 2 — Effect of LL-37 on bactericidal activity of serum against N. gonorrhoeae. FA1090 gonococci were incubated for 2 or 3 hours with 10% of NHS+/-OmCI in the presence or in the absence of 0.8 μg/mL of LL-37 with 20 μg/mL of C4BP-IgM. Two-way ANOVA with Tukey´s multiple-comparison test was performed to analyze differences among samples within the same hour. Bars indicate mean+/-SD, and circles refer to 3 independent repeats. Survival of bacteria was analyzed as Log(CFU/mL) and horizontal dotted line indicates the starting number of bacteria used in the assay. *p < 0.05, **p < 0.01, ***p < 0.005, ****p < 0.0001. [file Image_2.tiff]

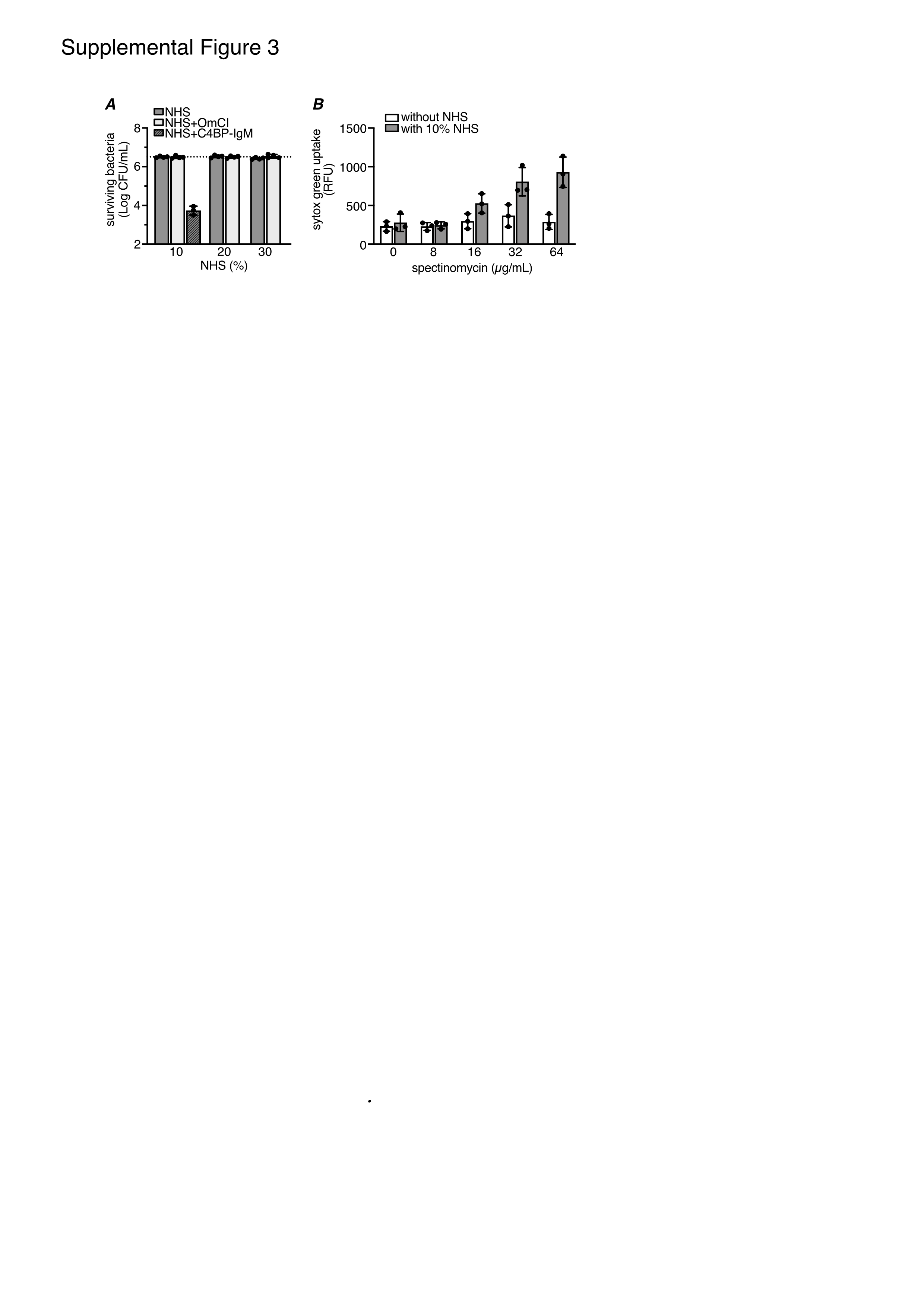

Supplement: Supplementary Figure 3 — FA1090 susceptibility to NHS or spectinomycin. (A) FA1090 gonococci were incubated for 30 min at 37°C with increasing concentrations of NHS, and survival was estimated as Log(CFU/mL) with horizontal dotted line indicates the starting number of bacteria used in the assay. Sample with 10% NHS + C4BP-IgM (10 μg/mL) is used as a positive control for bactericidal activity of serum. (B) FA1090 gonococci were incubated for 3 hours with or without 10% NHS and increasing concentrations of spectinomycin. Internalization of sytox green was measured after 30 min incubation. In all graphs, bars indicate mean+/-SD, and circles show 3 independent repeats. [file Image_3.tiff]

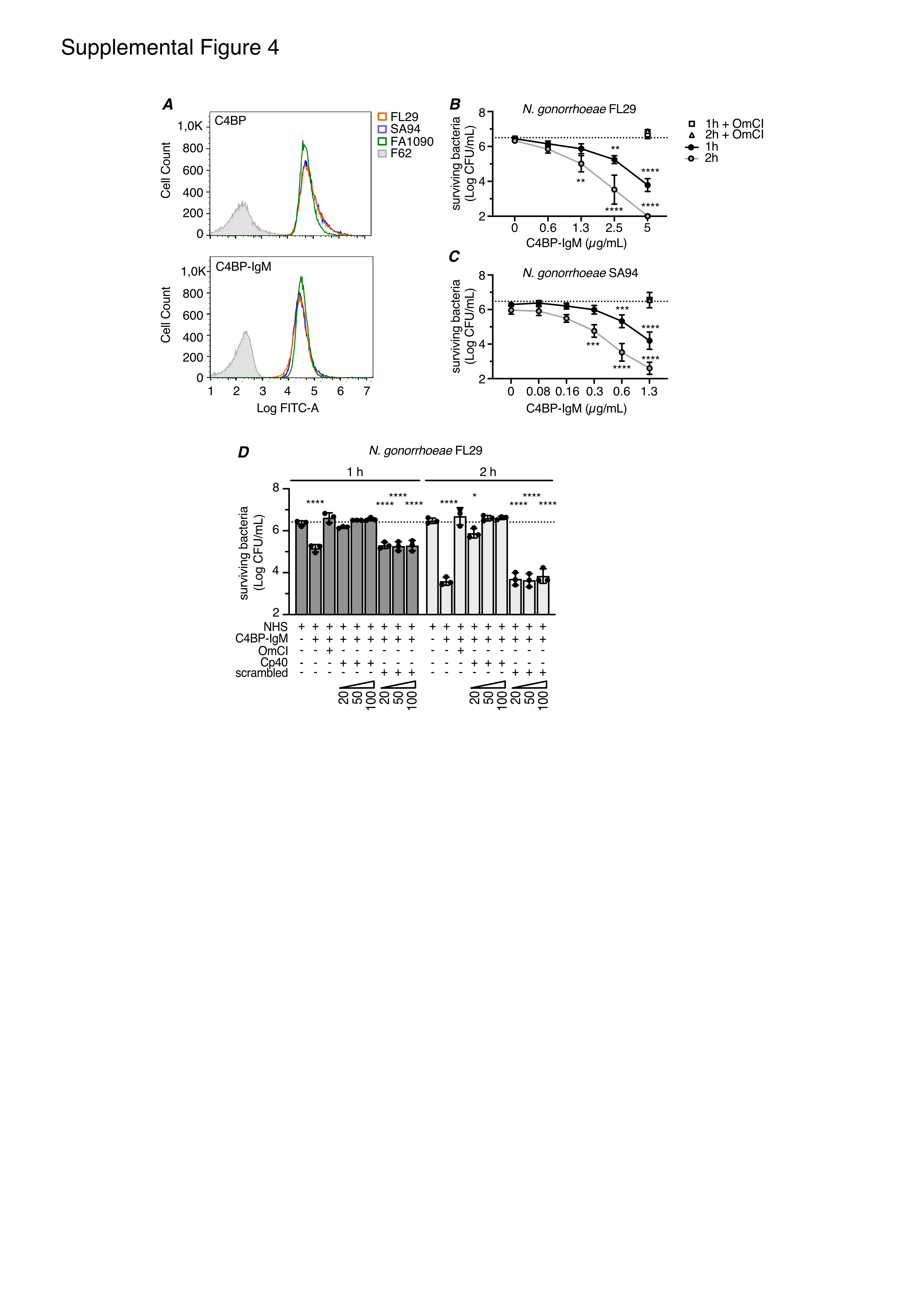

Supplement: Supplementary Figure 4 — Azithromycin resistant strains of N. gonorrhoeae. (A) Flow cytometric determination of binding of fluorescently labeled C4BP and C4BP-IgM to FL29 and SA94 gonococci after 30 min of incubation at 37°C. FA1090 and F62 were used as controls for positive and negative binding, respectively.(B, C) FL29 and SA94 gonococci were incubated for 1 or 2 hours with increasing concentrations of C4BP-IgM in the presence of 10% NHS+/-OmCI. Circles display mean+/-SD of 3 independent experiments. At each time point, differences between samples with C4BP-IgM and those without the protein were compared using two-way ANOVA with Dunnett´s multiple comparisons test. (D) FL29 gonococci were incubated for 1 or 2 hours with 10% NHS+/-OmCI or increasing concentrations of Cp40 or scrambled peptide (10, 50 or 100 μM) in the presence or in the absence of 2.5 μg/mL of C4BP-IgM. Scrambled peptide is used as irrelevant protein for Cp40-treated samples. Two-way ANOVA with Dunnett´s multiple comparisons tests was performed considering sample with NHS alone as reference. Bars display mean+/-SD with circles indicating independent repeats; n = 3. Horizontal dotted line indicates the starting number of bacteria used in the assay. *p < 0.05, **p < 0.01, ***p < 0.005, ****p < 0.0001. [file Image_4.tiff]
